# Effect of Buffer on Protein Stability in Aqueous Solutions: A Simple Protein Aggregation Model

**DOI:** 10.1021/acs.jpcb.0c10339

**Published:** 2021-03-03

**Authors:** Sandi Brudar, Barbara Hribar-Lee

**Affiliations:** Faculty of Chemistry and Chemical Technology, University of Ljubljana,, Večna pot 113, SI-1000 Ljubljana, Slovenia

## Abstract

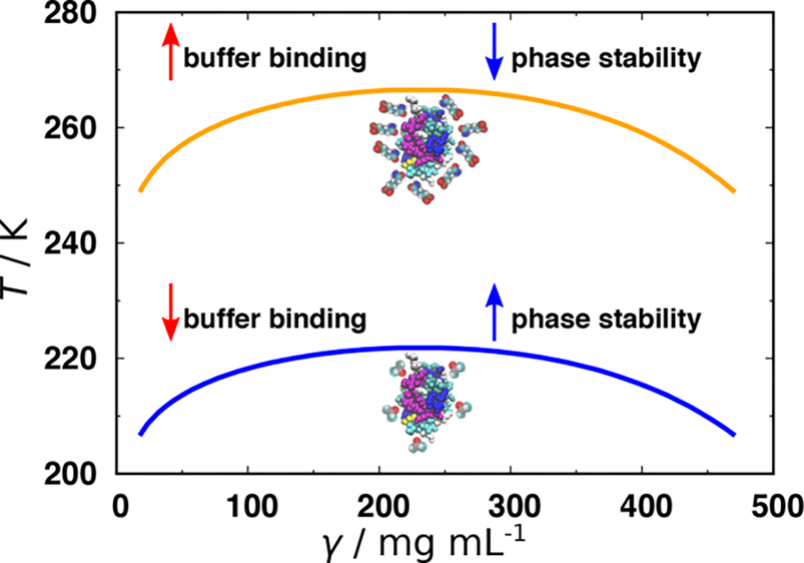

Liquid–liquid phase separation (LLPS) of proteins has recently been associated with the onset of numerous diseases. Despite several studies in this area of protein aggregation, buffer-specific effects always seem to be overlooked. In this study we investigated the influence of buffers on the phase stability of hen egg-white lysozyme (HEWL) and its respective protein–protein interactions by measuring the cloud point temperature, second virial coefficient, and interaction diffusion coefficient of several HEWL–buffer solutions (MOPS, phosphate, HEPES, cacodylate) at pH 7.0. The results indicate that the buffer molecules, depending on their hydration, adsorb on the protein surface, and modulate their electrostatic stability. The obtained information was used to extend the recently developed coarse-grained protein model to incorporate buffer-specific effects. Treated by Wertheim’s perturbation theory the model qualitatively correctly predicted the experimentally observed phase separation of all investigated HEWL–buffer solutions, and further allowed us to predict the phase stability of protein formulations even in experimentally unattainable conditions. Since the theory can be straightforwardly extended to include multiple components it presents a useful tool to study protein aggregation in crowded cell-like systems.

## Introduction

Proteins have an indisputable role in key life processes of all organisms. They are the most abundant biomolecules in living cells. Furthermore, they often appear in pharmaceutical and biotechnological applications as antibodies in vaccines, delivery systems, and as other biological drugs. In order to maintain all of their functions proteins have to remain stable in their natural environment, usually an aqueous solution. Interparticle interactions are those that dictate the thermodynamic stability of aqueous protein solutions and can sometimes lead to changes in protein structure and/or conformation which results in the loss of protein function. The thermodynamic instability of these solutions, on the other hand, is reflected in the formation of the two-phase region in otherwise homogeneous protein solutions.

In multicomponent systems, such as biological cells, if we gradually decrease the temperature of the system at sufficient protein concentration, liquid droplets of high protein concentration start to form. Soon these droplets merge to form the protein-rich phase that separates from the rest of the solution due to gravity. This process is known as the liquid–liquid phase separation (LLPS). Despite the vast number of different proteins and solution conditions in various applications most globular proteins exhibit similar phase behavior: the liquid–liquid coexistence curve is located at temperatures lower than the crystallization solubility line, and is often substantially broader than predicted by the mean-field theory, the feature originating from the intrinsic short-range, and anisotropic nature of the protein–protein interactions.^1^ Although the protein-rich phase can be generally useful for crystallization purposes (due to formation of glassy solids), it can also cause severe damage due to forming insoluble protein aggregates, among which predominate highly ordered amyloid fibrils.^2^ All these insoluble aggregates can cause a number of neurodegenerative diseases (e.g., Alzheimer’s, Parkinson’s, and Huntington’s disease, etc.).^3^

The need for understanding this protein phase behavior is 2-fold: in the pharmaceutical industry preparing stable liquid formulation of therapeutic biologics is of critical importance. At the same time comprehending the factors leading to the LLPS in protein solutions would contribute to better understanding of cell organization where the formation of biomolecular condensates is critical to maintain the biochemical reactions essential for life.^2,4,5^

Since protein interactions are governed by many factors, such as pH, surface hydrophobicity, surface charge distribution, salt and buffer type, ionic strength etc. the phase behavior of protein solutions is still not well understood.^6^ Further, biological mixtures, such as cytosols may consist of thousands of distinct components, and prediction of phase stability of such multicomponent systems is only possible using coarse-grained models. Even with those, computer simulations that represent a popular choice to study the microscopic picture of processes in solution can be demanding and often, due to statistical error cannot lead to reliable conclusions.^7^

Recently a coarse-grained protein model has been proposed that, coupled with Wertheim association thermodynamic perturbation theory, successfully predicted the phase stability of simple proteins and antibodies in aqueous salt solutions.^8,9^ Here we propose the extension of the model to include the buffer-specific effects that have been shown to have a substantial influence on the protein–protein interactions.^10−13^ This would allow us to predict phase stability of protein formulations even in the range of temperatures and concentrations where the measurements are very difficult or impossible to obtain. However, the theory is currently still incapable to reproduce another important feature of protein aggregation–amyloid fibril formation.^2,14,15^

To include the buffer specificity into the model we first experimentally determined the influence of different buffers on the protein–protein interaction. Due to its robustness and relatively good solubility over a broad range of conditions, lysozyme (HEWL in particular) has been chosen for these studies.

The paper is organized as follows. After this Introduction, we briefly describe the experimental and theoretical methods used. The Results and Discussion section describes our experimental observations, and the incorporation of buffer-specific effects into the model. The Conclusions are presented at the end.

## Materials and Methods

### Materials

Hen egg-white lysozyme (HEWL), sodium dihydrogen phosphate dihydrate, sodium bromide, sodium hydroxide, glycine, acetic acid, Amicon Ultra-15 centrifugal units, Spectra/Por float-a-lyzer G2 dialysis tubes and hydrochloric acid were purchased from Merck (Darmstadt, Germany). Cacodylic acid, HEPES, MOPS, and TRIS were obtained from Sigma-Aldrich (St. Louis, U.S.A.). Disodium hydrogen phosphate was purchased from Chem-Lab (Zedelgem, Belgium).

### Buffers and NaBr

Various buffers with an ionic strength of 0.1 M were prepared to study how buffers affect phase stability of aqueous HEWL solutions. To avoid denaturation of HEWL, we chose a pH value of 7.0, which is close to physiological conditions and is applicable to a broad range of buffers. Desired pH of a buffer solution was obtained by adding appropriate quantities of 1 M hydrochloric acid or sodium hydroxide to it. All buffers were filtered through 0.45 μm filter pores (Sartorius) before use. NaBr was first dried for 3 h in the presence of P_2_O_5_ at 107 °C. NaBr was then added to all buffer solutions in way to create stock salt-buffer solutions with a two times higher salt concentration than later intended for cloud point measurements. The pH of salt-buffer solutions was also examined and corrected with 1 M hydrochloric acid or sodium hydroxide to obtain same values as for pure buffers.

### HEWL Solutions

We prepared different combinations of HEWL-buffer solutions, depending on the type of experiment performed. The protein and salt concentration ranges were selected in a way to still preserve the native structure of HEWL and at the same time be able to reach its phase separation. The direct protein–protein interactions were examined in the low concentration regime (*c* < 10 mg/mL), while the liquid–liquid phase separation was studied in the intermediate to high concentration regime in which protein aggregation may occur (40 mg/mL < *c* < 200 mg/mL). For cloud-point measurements stock HEWL-buffer solutions of 200 and 270 mg/mL of protein were prepared. Meanwhile, 100 mg/mL HEWL-buffer stock solutions were enough for dynamic light scattering (DLS) measurements. Protein concentrations were determined spectrophotometrically using the HEWL extinction coefficient of 2.64 mL mg^–1^ cm^–1^ at 280 nm.^16^ First, HEWL was readily dissolved in a chosen buffer. The obtained HEWL-buffer solution was then extensively dialyzed against the corresponding buffer at room temperature (three changes of buffer solution within 24 h) using a 5 mL Float-A-Lyzer dialysis tube with a 3500 Da cutoff. As the protein-buffer solutions were diluted during dialysis, they were all subsequently concentrated with 15 mL Amicon Ultra-15 centrifugal units at 5000 rpm and 4 °C. When HEWL concentrations of 180 and 250 mg/mL (2× higher than during measurement) were achieved, the protein-buffer solutions were filtered through 0.45 μm filter pores (Sartorius) to remove any remaining impurities.

### Cloud-Point Measurements

The cloud point temperature, *T*_cloud_, is defined as the temperature where the protein solution undergoes phase separation into two coexisting liquid phases. In established experimental procedures, *T*_cloud_ denotes the temperature at which, upon cooling, the first opacification of the protein solution occurs. In our study *T*_cloud_ for all samples were measured by Cary 100 Bio spectrophotometer (Varian, Australia).

#### Cary 100 Bio Spectrophotometer

NaBr-buffer solutions were filtered through 0.2 μm filter pores (Sartorious) and preheated between 40 and 50 °C. HEWL-buffer and NaBr-buffer solutions were mixed together in a 1:1 ratio just before the measurement and then transferred into black-walled quartz cuvettes with a path length of 1 cm and volume of 1 mL. The final concentration of HEWL was 90 and also 125 mg/mL, meanwhile NaBr concentrations ranged between 0.1 and 0.5 M. Afterward samples were subsequently cooled from 40 °C to around −6 °C, with a cooling rate of 0.1 °C/min. In order to prevent condensation on cuvette walls, a constant flow of dry nitrogen was provided during cooling. In our study the sample turbidity accompanying phase transition was detected as an increase in measured sample absorbance at 340 nm.

### Dynamic Light Scattering

Dynamic light scattering (DLS) measurements were carried out with the 3D-DLS-SLS cross-correlation spectrometer from LS Instruments GmbH (Fribourg, Switzerland). The source of incident light was a He–Ne laser with a wavelength of λ_0_ = 632.8 nm. HEWL-buffer solutions (from 4 to 70 mg/mL of HEWL) were prepared from 90 mg/mL stock solutions. Afterward, individual samples (approximately 2 mL) were filtered through 0.2 μm filters (Sartorius) directly into dust-free cylindrical glass cells and equilibrated inside the decalin chamber for 5 min before measurement. The light scattering measurement was performed at 90° and 25 °C. For each sample, ten correlation functions with a duration of 120 s were obtained. They were evaluated by the inverse Laplace transformation with the program UFORT (User Friendly Optimized Regularization Technique) to obtain the hydrodynamic radii, *R*_h_, of HEWL in different buffer solutions. In this way we also gained information on the corresponding diffusion coefficients, *D*, of HEWL from the following Stokes–Einstein equation:^17^
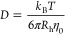
1where *k*_B_ is the Boltzmann constant and η_0_ the solvent viscosity. The interaction diffusion coefficient, *k*_D_, was obtained in the semidilute region of the concentration dependence of *D* using the following expression:
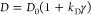
2where *D*_0_ is the self-diffusion coefficient of HEWL at infinite dilution and γ the HEWL mass concentration in mg/mL.

In order to obtain the second virial coefficient, *B*_22_, we first had to determine the scattered intensity of our samples, *I*_u,θ_^sam^, which can be calculated from the mean count rate (MCR) and laser intensity, *I*_laser_, as noted in eq 3:
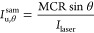
3To establish the Rayleigh ratios of samples, *R*_θ_^sam^, their scattering intensity was compared to the scattering intensity of a standard toluene solution, *I*_u,θ_^tol^, with a known Rayleigh ratio^18^*R*_θ_^tol^ = 14.0 × 10^–6^ cm^–1^
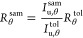
4Hence the *B*_22_ values of our samples were obtained from the semidilute region of the Debye plots constructed by using the following equation:
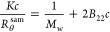
5where *M*_w_ is the average molecular weight of the protein and *c* its corresponding concentration in g/mL. While the optical constant *K* is defined as
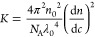
6where *n*_0_ is the solvent refractive index, *N*_A_ the Avogadro constant, and d*n*/d*c* describes the specific refractive index increment, which is for globular proteins 0.185 mL/g.^19^

### Wertheim’s Thermodynamic Perturbation Theory

According to the Wertheim’s perturbation theory of strongly associating liquids (TPT1),^20^ the potential between two proteins *u*(***r***) can be described as a sum of two contributions, namely the hard-sphere part *u*_R_(*r*) and the attractive contributions *u*_AB_, which arise from the short-range square-well interaction sites on the surface of the protein:
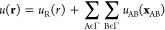
7where ***r*** (*r* = |***r***|) is the vector between the centers of two proteins, **x**_AB_ is the vector that connects interaction sites A and B on two different proteins, and Γ represents the set of independent sites. Since the sites are distributed over the surface of the protein molecules, their distance from the center of the spheres (*d*) can be written as *d* = σ/2. We do not distinguish between attractive sites, therefore the associative potential *u*_AB_ is equal among all sites. The pairwise additive potential can then be expressed by
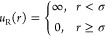
8
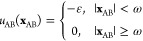
9where ε (ε > 0) is the square-well potential depth and ω its corresponding range. Hence, the interaction between particles only takes place when the distance between two sites |**x**_AB_| is within the pair potential range ω. We also incorporate the premise, according to Wertheim,^20,21^ that multiple site bonding is prohibited by taking into account the following expression:
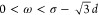
10Hereafter, we can assume the additivity of the Helmholtz free energy of our system:
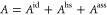
11where *A*^id^ and *A*^hs^ are the ideal and hard-sphere contributions, respectively,^22^ while *A*^ass^ denotes the free energy contribution due to site–site interactions. This association contribution can be written according to TPT1,^21,23,24^ as follows:
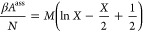
12where β = 1/*k*_B_*T* and *k*_B_ is the Boltzmann’s constant. Meanwhile *X* denotes the average fraction of monomers in the system that are not bonded to any site and can be obtained from the mass-action law:^23^
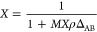
13The association parameter Δ_AB_ can be determined in the so-called sticky limit:^21^
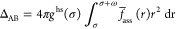
14One can calculate the contact value for the radial distribution function of a fluid of hard-spheres *g*^hs^(σ) from the Ornstein–Zernike integral equation theory employing the Percus–Yevick (PY) closure,^25^ which gives
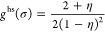
15where η is the packing fraction, related to density as η = *π ρσ*^3^/6. Meanwhile, *f̅*_ass_(*r*) is the angular average of the Mayer function, which can be obtained analytically as^8,21^
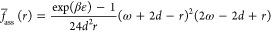
16Once we obtain the Helmholtz free energy of our system, we can compute other thermodynamic quantities, among them the osmotic pressure Π and chemical potential μ, through standard thermodynamic relations:
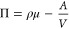
17
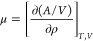
18
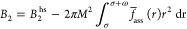
19where *B*_2_^hs^ = 2*πσ*^3^/3 is the hard-sphere contribution to the second virial coefficient.^26^

### Viscosity of Buffers

Viscosities of all buffers at pH 7.0 were measured with the ViscoSystem AVS 370, which is equipped with a LAUDA DLK 10 prethermostat and a LAUDA Eco Silver main thermostat. Aqueous buffer solutions at different concentrations (from 0.02 to 0.1 M) were individually transported (2 mL of solution) into the Ostwald viscometer (Micro-Ostwald V4 Kap I 51710 A) and thermostated for 10 min prior measuring the time of flow of samples at 5 and 25 °C. The final time of flow was obtained as an average of five measurements. To determine the viscosities of samples we also had to determine the densities of all buffer solutions by using a six-digit accurate Anton-Paar DMA 5000 density meter. Approximately 1.5 mL of each buffer solution was loaded into the U-tube of the density meter, with special care taken not to insert air bubbles that could interfere the measurement. At identical conditions as buffer samples we also measured the time of flow and density of milli-Q water. To obtain the viscosities of all buffer solutions we needed the viscosity of water at 5 and 25 °C, which were taken from Huber et al.^27^ and were found to be 1.5182 and 0.8900 mPa s, respectively. The viscosities of buffers were then calculated from the following equation:
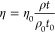
20where η, ρ, and *t* are the viscosity, density, and time of flow of investigated buffers, respectively. Meanwhile η_0_, ρ_0_, and *t*_0_ are the same parameter values for water. To determine the Jones–Dole B coefficient of buffer ions, which can be used to classify ions as structure-makers (kosmotropes) or structure-breakers (chaotropes), we applied the Jones–Dole equation,^28^ which can be written as
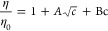
21where *A* is a coefficient that describes the influence of charge–charge interactions on the viscosity of the sample and can be obtained from Debye–Hückel theory. *B* denotes the Jones–Dole coefficient, which illustrates the solute–solvent interactions at a given temperature. Meanwhile *c* represents the solute concentration. Parameters *A* and *B* were obtained from the best fit of eq 21 to the experimentally measured relative viscosity (η/η_0_) at different solute concentrations.

## Results and Discussion

### Experimental Characterization of Protein–Protein Interactions in HEWL Solutions

Two experimental indicators commonly used to probe the interactions in protein solutions are the second virial coefficient, *B*_22_, and the diffusion interaction parameter, *k*_D_, both known to reflect the strength of the protein–protein interaction. While both quantities have been determined before for lysozyme solutions as a function of added salt concentration,^4,29−31^ we here focused on these quantities as a function of the buffer specificity. Most of chosen buffers are present as anionic species (molecular structure shown in Figure S6) and contain sodium cations as counterions. The effect of different counterions was not tested in this study since it has been previously observed that the nature of cations has only a minor effect on the stability of positively charged proteins.^32,33^ The values for both quantities that were determined from the semidilute protein regime (see Figure S2) in different buffer solutions at 298 K and zero additional salt present are listed in Table 1. Both sets of quantities clearly depend on the buffer identity, indicating the role of buffer molecules in modifying protein–protein interactions. An interesting observation is that even though the temperature 298 K is above the critical temperature in all cases studied,^33−35^ negative values for *B*_22_ and *k*_D_ coefficient were obtained in phosphate buffer, indicating net attractive interaction between protein molecules.^30,36^ One possible explanation for this is that due to the high charge density of phosphate ions present in phosphate buffer these ions interact with positively charged amino acid residues on protein surface and screen the repulsion between protein molecules, enabling them to come closer together.^30,36,37^

**Table 1:** List of Measured *B*_22_ and *k*_D_ Values of HEWL in Chosen 0.1 M Buffers with pH 7.0

buffer	*k*_D_ (mL g^–1^)	*B*_22_ (×10^–4^ mol mL *g*^–2^)
MOPS	11.0 ± 2.0	5.9 ± 0.2
HEPES	13.5 ± 0.5	2.0 ± 2.2
cacodylate	12.0 ± 1.0	3.1 ± 0.2
phosphate	–7.1 ± 0.8	–1.2 ± 0.2

To further inspect the role of buffers in modifying protein–protein interactions we determined the cloud-point temperatures^38^ of HEWL in different buffer solutions. Only minor changes in *p*H are expected in this temperature range^39,40^ which, however, are not sufficient to affect the aggregation properties of HEWL under these conditions.^15^

**Figure 1:**
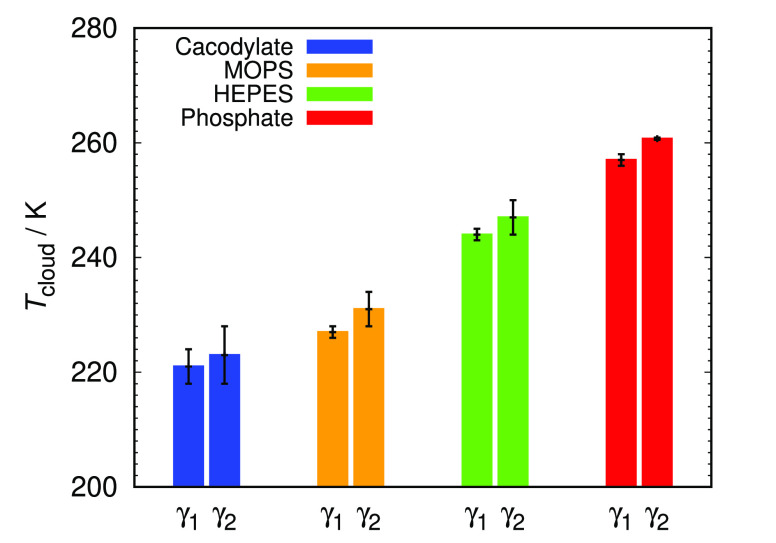
Measured *T*_cloud_ values of HEWL at two concentrations in chosen 0.1 M buffers with pH 7.0 extrapolated to zero NaBr concentration, where γ_1_ and γ_2_ denote 90 and 125 mg/mL HEWL, respectively.

Since in all cases studied the temperature of phase separation was substantially below zero degrees where the experimental setup did not enable us to obtain meaningful results, we have used the extrapolation method where the cloud point was determined at different NaBr salt concentration, and the data were then extrapolated to zero salt concentration (see Figure S1).^34^ The results for the cloud-point temperature in different buffer solutions and at two different protein concentrations are shown in Figure 1.

**Figure 2:**
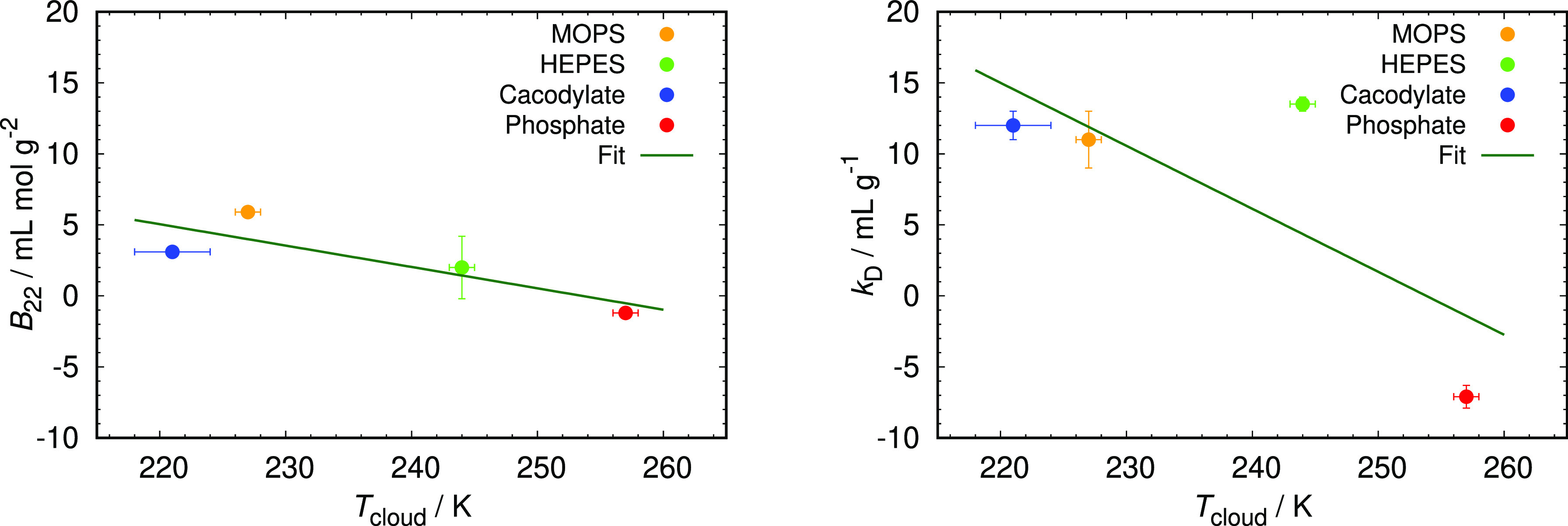
Correlations between measured *B*_22_ and *k*_D_ with *T*_cloud_ values for 90 mg mL^–1^ of HEWL in selected 0.1 M buffers with pH 7.0. The straight line was obtained with a best linear least-squares fit to the experimental data.

One can notice significant differences among *T*_cloud_ values for buffers at an identical pH value of 7.0, thus indicating to buffer-specific effects. When comparing the *T*_cloud_ at two different HEWL concentrations one can notice that the opacification of solutions containing more HEWL occurs at slightly higher temperatures, which is in good agreement with the established course of the HEWL phase diagram.^34,35,41^ Based on the obtained *T*_cloud_ values the highest HEWL phase stability is established in cacodylate buffer, and the lowest phase stability in phosphate buffer that was already indicated by negative *B*_22_ and *k*_D_ values. Even for other buffers studied, *T*_cloud_ shows high correlation with second virial coefficient, as well as with the diffusion interaction parameter (Figure 2).

With the exception of HEPES for *k*_D_ that somewhat deviates from the rest, an approximately linear correlation of both *B*_22_ and *k*_D_ with *T*_cloud_ is observed, signifying that the more attractive interactions between HEWL molecules (more negative *B*_22_ and *k*_D_ values), the faster the destabilization of HEWL–buffer solutions upon cooling (higher *T*_cloud_). Similar observations on the correlation of *T*_cloud_ with second virial coefficient were also obtained by Platten et al. for HEWL in acetate buffer.^42^

### Modeling the Buffer-Specific Effects in Phase Transition

**Figure 3:**
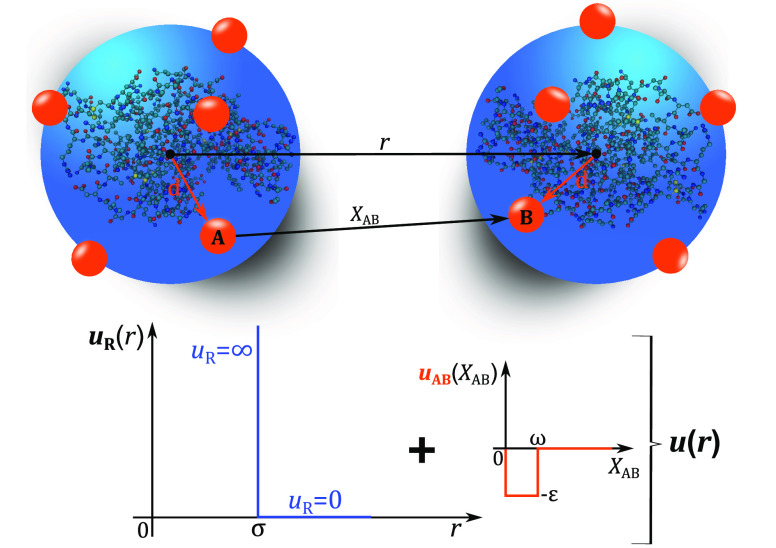
Coarse-grained model of HEWL with the shown sum of hard-sphere and attractive interactions between proteins.

In the last few decades it has been established that “the isotropic models fail to describe the phase diagram of protein solutions quantitatively and cannot address phenomena such as protein aggregation and self-assembly”.^43,44^ To predict the right shape of the liquid–liquid phase diagram, the protein–protein interactions have to be anisotropic in nature, and short-ranged.^1^ In the simple coarse-grained model used in this work we model the protein solution as a one-component system of protein molecules where, as proposed in Kastelic et al.,^8^ the protein molecules are represented as hard spheres of diameter σ with short-ranged attractive protein–protein interaction sites on the sphere surface (Figure 3).

The solvent (comprising of water, buffer, and simple salt ions) is treated on McMillan Mayer level of approximation as an effective modifier of the protein–protein interactions. As such, the protein–protein interaction potential can be given by eqs 7–9.^8^ In this model the square-well potential depth, ε that determines the attraction between two model protein molecules depends on the ionic strength, *I* of a simple salt in solutions as
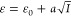
22where ε_0_ is the interaction parameter at zero additional simple salt present while parameter *a* depends on the nature of the salt.^37^ ε parameter as a whole has been determined to best reproduce the experimentally determined liquid–liquid phase diagram.^8^

As distinct from the original model described in,^8^ our simple model is determined by six parameters: protein diameter, σ, the corresponding protein molecular weight *M*_2_, ω which represents the range of the square-well potential, the number of binding sites on the spherical protein surface, *M*, and two interaction parameters ε, and *a*. Assuming the buffer influences mostly the protein–protein attractive interaction we fixed four of the parameters, and kept them same as in previous work of Kastelic et al.^8^ They are given in Table 2. This was further justified by the values of radius of hydration of lysozyme molecule determined by DLS measurements (given in Table S1) that show (within experimental error given in the SI) no significant dependence on the buffer in the solutions. The protein model radius used in this work was obtained from solvent excluded surface volume (SESV) for a given protein structure (1DPX for HEWL). Assuming that the protein is spherical, a volume of a sphere with this same SESV was calculated, from which radius of the model protein was derived.

**Table 2:** List of Optimal Parameters of the Spherical Protein Model for the Construction of Phase Diagrams of HEWL in Different Buffer Solutions

parameter	value
σ/nm	3.43
ω/nm	0.18
*M*	10
*M*_2_/g mol^–1^	14300

**Figure 4:**
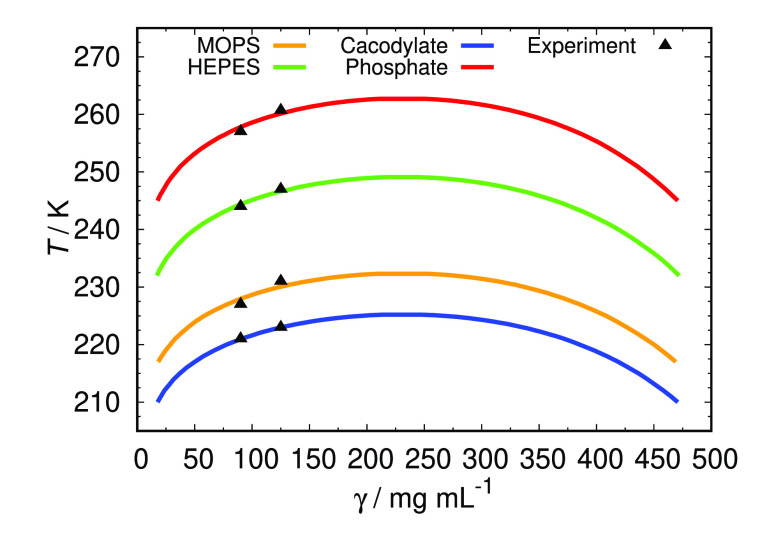
Comparison of predicted phase diagrams of HEWL in different 0.1 M buffers and pH 7.0 with experimentally obtained *T*_cloud_ values extrapolated to zero salt concentration.

The parameter ε_0_ was then determined in such a way to reproduce the experimentally determined cloud-point temperature, *T*_cloud_, at a single (e.g., 90 mg/mL) protein concentration at zero added salt. The ε_0_ are for all of the buffers used listed in Table 3. We used these values to calculate the whole liquid–liquid coexistence curve for each buffer used, and the results are shown in Figure 4. The shape of the calculated phase diagrams is qualitatively the same as obtained previously for HEWL solutions at somewhat different pH.^34^ To further test the validity of the obtained parameter at pH 7.0 we experimentally determined *T*_cloud_ also at somewhat higher protein concentration (125 mg/mL), the results for which are also presented in Figure 4. One can see that excellent agreement between theory and experiment is observed. The value of ε_0_ parameter reported by Janc et al.^37^ for HEWL in phosphate buffer solutions at same ionic strength, but somewhat lower pH (e.g., 6.8) is slightly higher (2293 K) which we can contribute to a difference in pH. Since the net surface charge of HEWL molecule increases with decreasing pH, stronger electrostatic interaction between protein and buffer molecules is expected, increasing the net attraction between two protein molecules. We also investigated the possibility of hydrogen bonding between HEWL and buffer molecules by docking buffer molecules in question to the HEWL surface using YASARA computational tools. However, we did not find any reliable interactions that could indicate hydrogen bonding between protein and buffer molecules.

**Table 3:** Values of Ion-Specific Salt Factor, *a*, and Square-Well Potential Depth at Infinite Salt Dilution, ε_0_, for Selected 0.1 M Buffers at pH 7.0

buffer	*a*/K L^1/2^ mol^–1/2^	ε_0_/*k*_B_/*K*
phosphate	500 ± 20	2250 ± 10
HEPES	630 ± 40	2140 ± 10
MOPS	890 ± 40	1990 ± 10
cacodylate	1090 ± 70	1940 ± 20

In the recent years more and more experimental evidence is emerging indicating that buffer molecules can specifically adsorb at the charged protein surfaces modifying the protein–protein interactions.^10,12^ This effect is for adsorbed simple ions to protein surface well investigated, and known as Hofmeister effect.^45−48^ It is been established that the propensity of different ions to precipitate proteins can be correlated to their hydration properties.^45,49−51^ We therefore examined the effect of buffer ions on the water structure by determining their Jones-Dole *B* viscosity coefficient. This quantity is known to define the degree of water structuring, and is positive for kosmotropic ions and negative for chaotropic ions.^52^ The results for Jones-Dole coefficients determined at 5 and 25 °C are given in Table 4.

**Table 4:** Jones-Dole B Coefficients for Different Buffers at pH 7.0 at Two Different Temperatures

	Jones-Dole *B* coefficient/L mol^–1^	
buffer	5 °C	25 °C
phosphate	0.47 ± 0.04	0.50 ± 0.02
HEPES	0.66 ± 0.06	0.60 ± 0.06
MOPS	0.79 ± 0.17	0.74 ± 0.13
cacodylate	0.84 ± 0.15	0.52 ± 0.05

All of the determined Jones-Dole coefficient values were positive, suggesting that the buffer ions show a certain degree of water ordering as a consequence of electrostatic potential around ions. The ions with larger Jones-Dole viscosity coefficient bind the neighboring water molecules stronger, while the ones with lower Jones-Dole viscosity coefficient could release their hydration water molecules more easily, being able to come to closer proximity of the protein, and adsorb on the protein surface oppositely charged amino-acid residues.^53^ By adsorbing on the surface, buffer ions reduce the charge on the protein molecules, and thus decrease their electrostatic stabilization.

**Figure 5:**
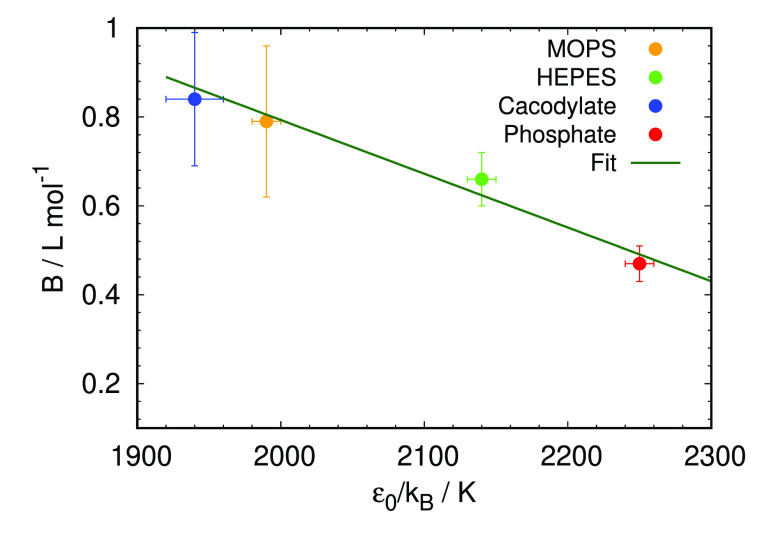
Correlation between Jones-Dole coefficients, *B*, and the square-well depth parameter at zero NaBr concentration, ε_0_/*k*_B_, for different 0.1 M buffers at pH 7.0.

The increased tendency of proteins to form clusters is reflected in larger attractive interaction parameter ε_0_. By plotting the dependence of Jones-Dole *B* coefficient on the strength of the interaction between proteins in the proposed model, ε_0_, we have obtained their linear relationship, as seen in Figure 5. The highest value of ε_0_ (indicating strongest attraction between proteins) was obtained for phosphate buffer that has at the same time the lowest Jones-Dole B coefficient (indicating more loosely bound water molecules), which results in the lowest phase stability of HEWL, meanwhile the lowest value of ε_0_ was determined for cacodylate, which has the strongest interaction with water, with HEWL demonstrating the highest phase stability under such conditions.

This trend of binding buffer ions to HEWL surface was further confirmed by measuring the zeta potential of different HEWL–buffer solutions (see Figure S3).

In the presence of salt the simple ions compete with buffer ions for adsorption on the protein molecule. We therefore examined the salt dependence of the model parameters in more details. The salt-specific parameter *a* from the eq 22 for NaBr salt has been determined by fitting the experimental data for cloud point temperature to the calculated quantity at both protein concentrations, e.g., 90 mg/mL, and 125 mg/mL, varying the strength of the interaction in the model, ε. The dependence of the ε parameter on the square-root of the salt concentration is shown in Figures S4 and S5 which display the application of eq 22 to all HEWL solutions in 0.1 M buffers at pH 7.0. We obtained a satisfying fit with theoretically obtained points that enabled us to determine parameters *a* and ε_0_, which are gathered in Table 3.

Neither values of *a* or ε_0_ were found to significantly alter with HEWL concentration (results not shown), but from their values in Table 3 it appears they are highly dependent on the choice of buffer solution. Despite the fact that one would at first expect the salt-specific parameter *a* to be buffer independent, Table 3 shows its clear correlation with parameter ε_0_, which is directly related to pure buffer solutions (*c*_salt_ = 0).

**Figure 6:**
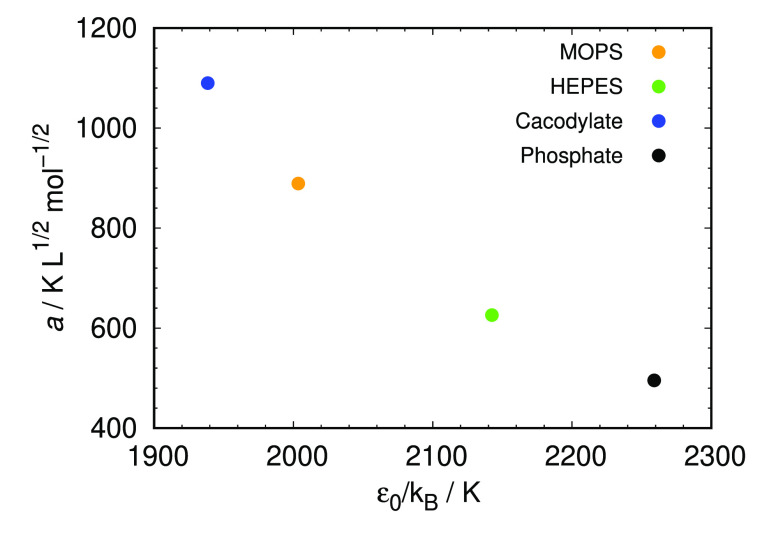
Correlation between the average salt-specific parameter *a* and the average square-well depth parameter at zero NaBr concentration, ε_0_.

The mutual relation between parameter *a* and ε_0_ is plotted in Figure 6. Figure 6 shows that the parameter *a* decreases when parameter ε_0_ increases. In other words, an increased presence of buffer ions on the surface of HEWL reduces the influence of salt ions (Br^–^) on effective protein–protein interactions. This result confirms our assumption about the competition of salt and buffer ions for binding to the surface of HEWL molecules.

## Conclusions

Different experimentally determined parameters, that are commonly used to evaluate protein–protein interactions in solution, such as second virial coefficient, and interaction diffusion coefficient were shown to depend on the buffer in which the protein solution is prepared, even at the same pH, and same ionic strength of the solution. A closer examination of the buffer properties indicated that the buffer ions bind to the oppositely charged amino-acid residues on the protein surface and in this way reduce the surface charge of the protein molecules that is one of the factors determining the stability of protein formulations. If other simple ions (salt) are also present in the solution, they compete with the buffer ions in the adsorption process. The propensity of buffer ions to adsorb is directly correlated to their Hofmeister properties.

Previously proposed simple coarse-grained model that can be successfully used to predict the phase stability of globular proteins has been extended to incorporate buffer-specific effects. Buffer molecules, as well as simple ions present in the solution modulate the attractive interaction between protein molecules through adsorption on the protein surface. The attractive interaction parameter can be split into the contribution of buffer molecules and contribution of simple ions, the importance of each depending on their hydration properties.

Even though a similar study has to be carried out with other proteins to obtain a more general relation between the attractive interaction parameter, and the identity of the buffer, our results clearly point to the importance of the buffer-specific effects on the stability of protein solutions and extend the applicability of the simple protein model to various solution conditions. Since the theory can be relatively straightforwardly extended to explicitly include other components in the model it could present a useful tool to predict protein aggregation in crowded multicomponent systems.

## Supporting Information Available

Additional information on the size of HEWL in different buffers; details of obtaining *T*_cloud_ in pure buffers; fitting of raw data of *k*_D_ and *B*_22_ measurements; data on zeta potential measurements; protein concentration dependence of some model parameters and the molecular structure of used buffers (PDF)
