# Supplementary material for: Effect of Buffer on Protein Stability in Aqueous Solutions: A Simple Protein Aggregation Model
Source: J Phys Chem B. 2021 Mar 3;125(10):2504–12. doi: 10.1021/acs.jpcb.0c10339 (PMC8041305; doi:10.1021/acs.jpcb.0c10339)
Supplement: Supplementary file 1 — jp0c10339_si_001.pdf [file jp0c10339_si_001.pdf]

# Supporting Information

## The Effect of Buffer on Protein Stability in Aqueous Solutions - a Simple Protein Aggregation Model

Sandi Brudar and Barbara Hribar-Lee*

*University of Ljubljana, Faculty of Chemistry and Chemical Technology, Večna pot 113, SI-1000 Ljubljana, Slovenia*

E-mail: barbara.hribar@fkkt.uni-lj.si

Table S1: $R_\mathrm{h}$ values of HEWL in different 0.1 M buffers at $p$H=7.0, based on the extrapolation of HEWL diffusion properties in the low protein concentration regime to infinite dilution. The errors were determined based on the experimental errors of measuring the diffusion coefficient of HEWL using DLS.

| Buffer | $R_\mathrm{h}$ / nm |
|---|---|
| Cacodylate | 2.29 ± 0.09 |
| Phosphate | 2.14 ± 0.02 |
| MOPS | 2.14 ± 0.03 |
| HEPES | 2.06 ± 0.04 |

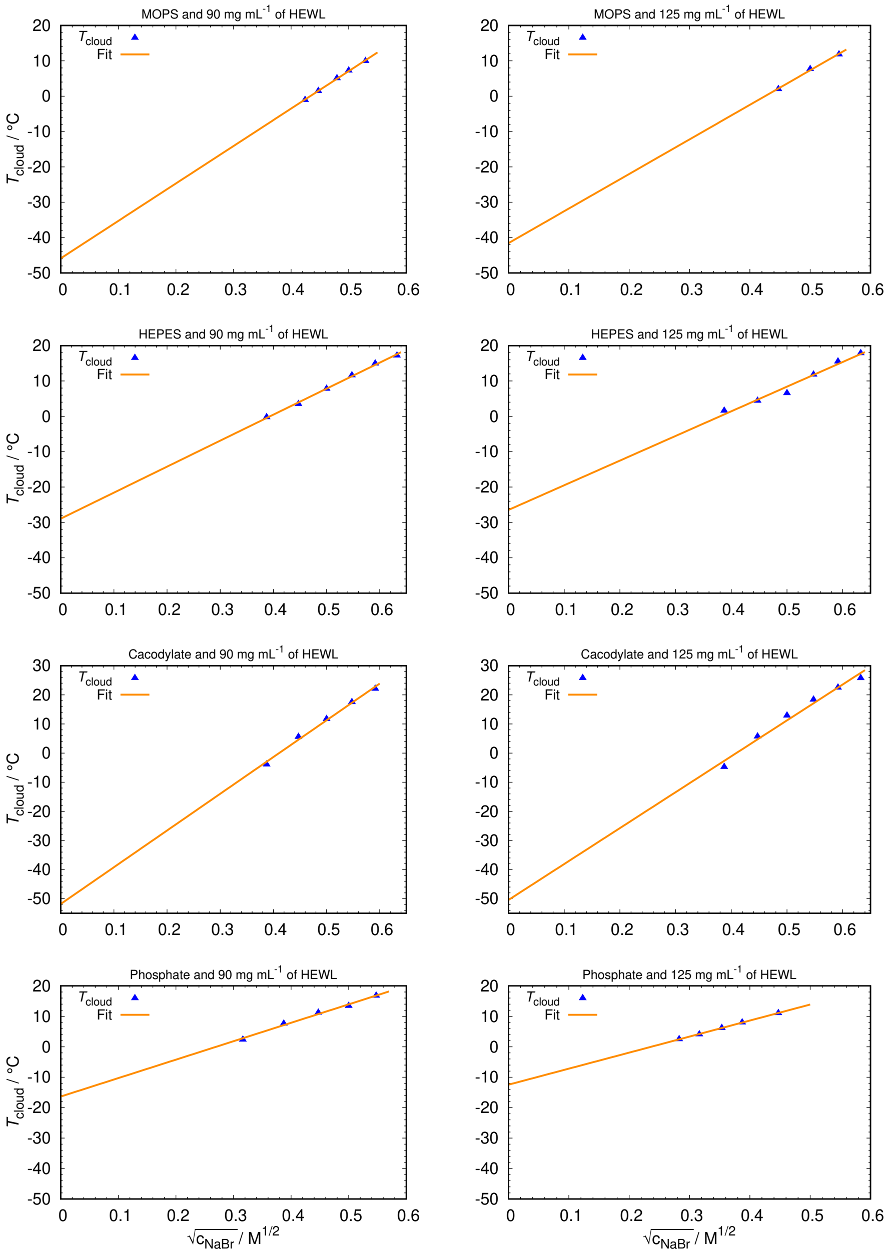


Figure S1: Least squares fit of $T_{\mathrm{cloud}}$ dependence on square root of concentration of added NaBr in chosen 0.1 M buffers at $p$H=7.0

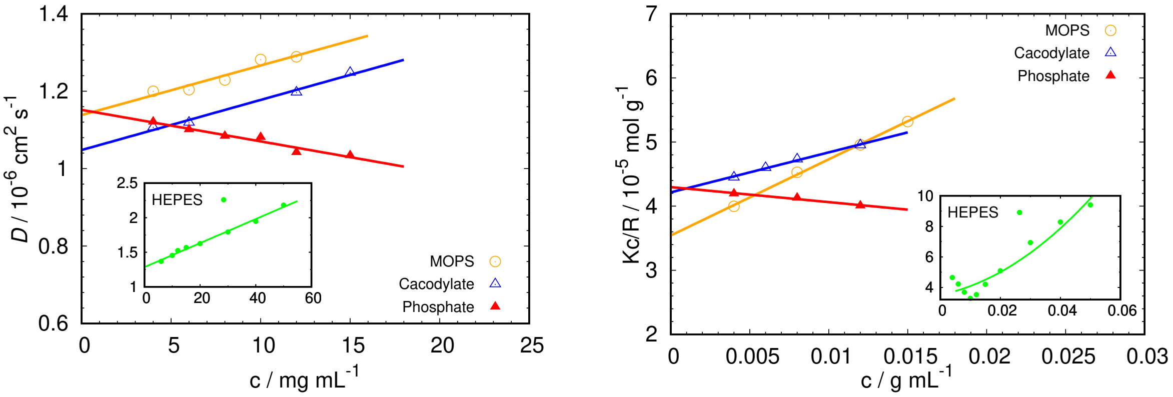


Figure S2: Least squares fit of diffusion coefficient dependence on HEWL concentration (left) and the Debye plot (right) in different 0.1 M buffers with $p$H=7.0 from which respective $k_\mathrm{D}$ and $B_{22}$ values were obtained. HEPES is displayed separately in the inset figure due to being fitted also in the high concentration range.

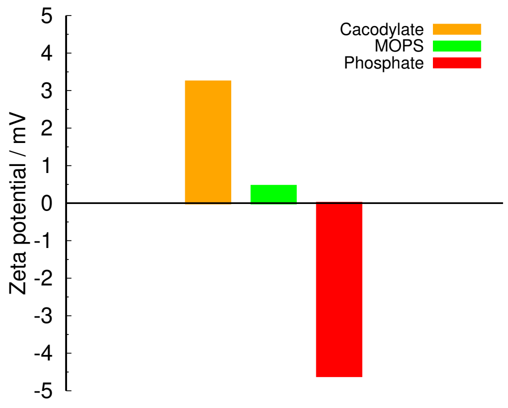


Figure S3: Zeta potential of 5 mg mL$^{-1}$ of HEWL in different 0.1 M buffers at $p$H=7.0. The observed trend confirms our hypothesis of binding of buffer ions on HEWL surface. The amount of bound buffer on HEWL surface increases from cacodylate, through MOPS and is the highest in the case of phosphate buffer.

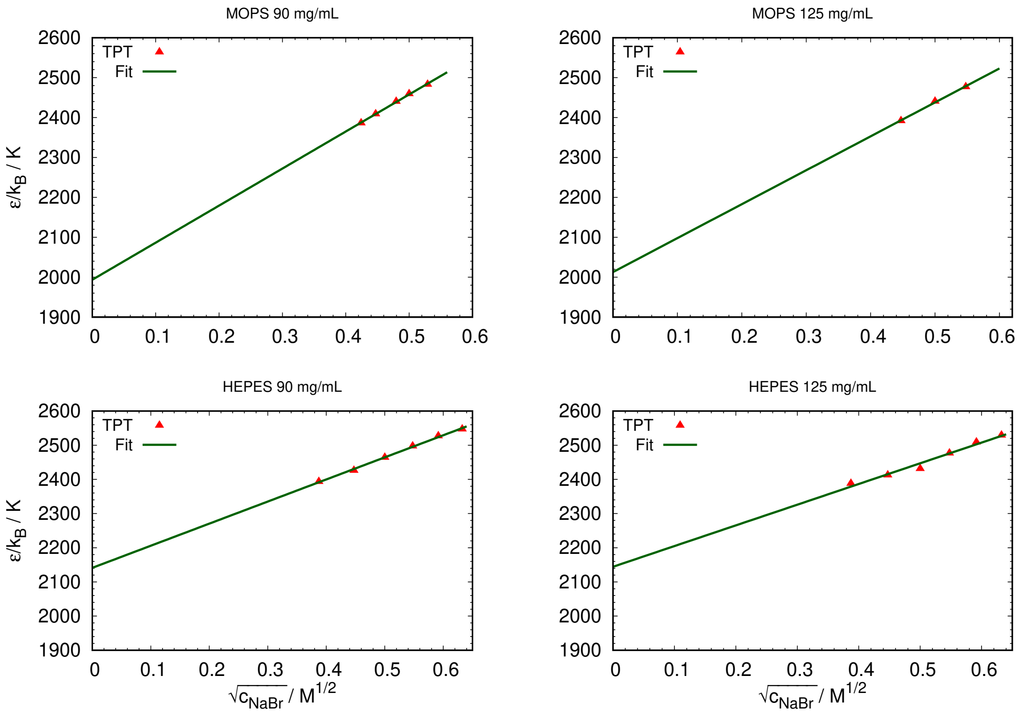


Figure S4: Least squares fit of theoretically obtained square-well potential depth dependence on square root of concentration of added NaBr in different 0.1 M buffers with $p$H=7.0 at 90 and 125 mg/mL of HEWL.

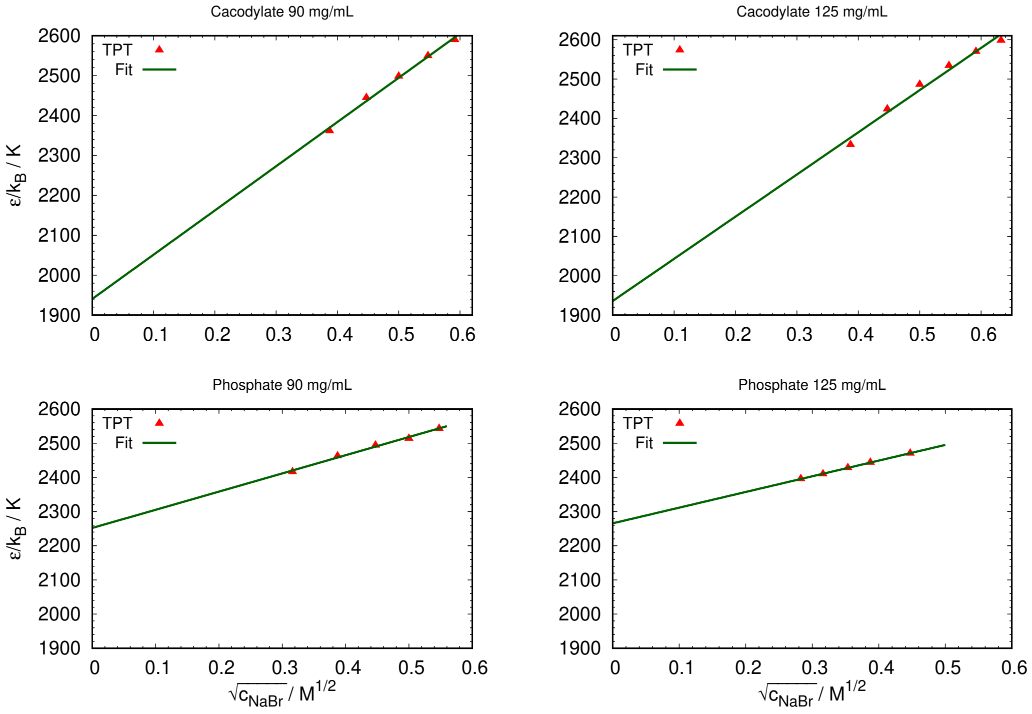


Figure S5: Least squares fit of theoretically obtained square-well potential depth dependence on square root of concentration of added NaBr in different 0.1 M buffers with $p$H=7.0 at 90 and 125 mg/mL of HEWL.

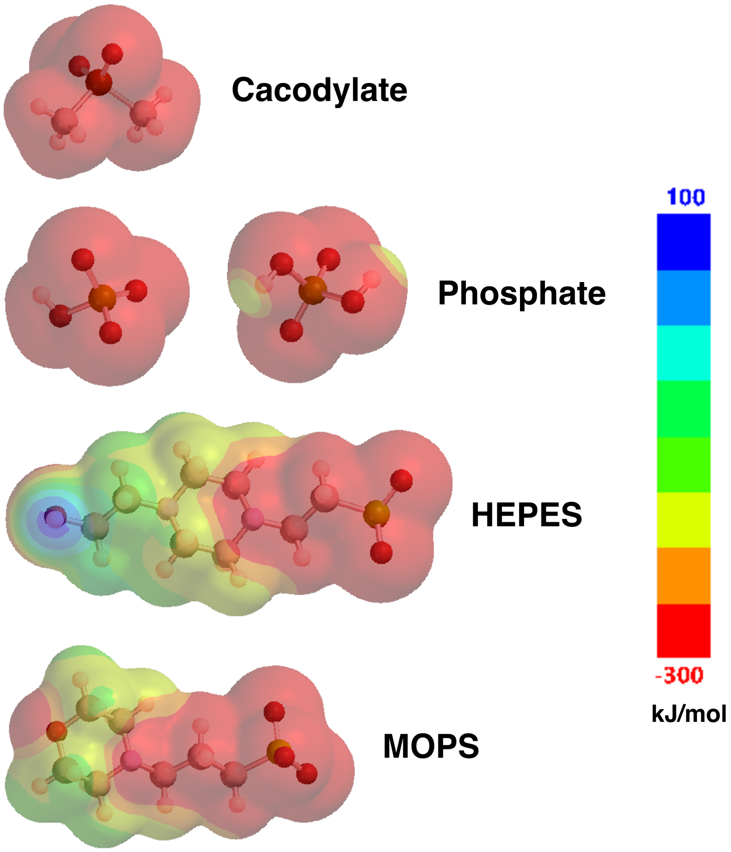


Figure S6: The molecular structure of buffers involved in this study with displayed surface electrostatic potential map.
